# ‘I wish someone watched me interview:’ medical student insight into observation and feedback as a method for teaching communication skills during the clinical years

**DOI:** 10.1186/s12909-016-0813-z

**Published:** 2016-11-09

**Authors:** Heather Schopper, Marcy Rosenbaum, Rick Axelson

**Affiliations:** 1 University of Iowa Carver College of Medicine, 375 Newton Rd, Iowa City, IA USA; 2 Department of Family Medicine and Office of Consultation and Research in Medical Education, 1204 Medical Education Building, University of Iowa Carver College of Medicine, Iowa City, IA USA; 3 Office of Consultation and Research in Medical Education, 1204 Medical Education Building, University of Iowa Carver College of Medicine, Iowa City, IA USA

**Keywords:** Communication skills, Undergraduate medical education, Clinical teaching, Student perspectives

## Abstract

**Background:**

Experts suggest observation and feedback is a useful tool for teaching and evaluating medical student communication skills during the clinical years. Failing to do this effectively risks contributing to deterioration of students’ communication skills during the very educational period in which they are most important. While educators have been queried about their thoughts on this issue, little is known about what this process is like for learners and if they feel they get educational value from being observed. This study explored student perspectives regarding their experiences with clinical observation and feedback on communication skills.

**Methods:**

A total of 125 senior medical students at a U.S. medical school were interviewed about their experiences with observation and feedback. Thematic analysis of interview data identified common themes among student responses.

**Results:**

The majority of students reported rarely being observed interviewing, and they reported receiving feedback even less frequently. Students valued having communication skills observed and became more comfortable with observation the more it occurred. Student-identified challenges included supervisor time constraints and grading based on observation. Most feedback focused on information gathering and was commonly delayed until well after the observed encounter.

**Conclusions:**

Eliciting students’ perspectives on the effect of observation and feedback on the development of their communication skills is a unique way to look at this topic, and brings to light many student-identified obstacles and opportunities to maximize the educational value of observation and feedback for teaching communication, including increasing the number of observations, disassociating observation from numerically scored evaluation, training faculty to give meaningful feedback, and timing the observation/feedback earlier in clerkships.

**Electronic supplementary material:**

The online version of this article (doi:10.1186/s12909-016-0813-z) contains supplementary material, which is available to authorized users.

## Background

Effective communication between physician and patient is critical to the delivery of quality medical care and as such teaching and evaluation of communication skills is an essential part of the clinical training of medical students [1, 2]. Despite the simplicity of this statement, there is real danger that students’ communication skills may decay during the clinical years because teaching is much more difficult to standardize than during pre-clinical years and student experiences vary greatly [3, 4]. The key to getting the most accurate and meaningful picture of what strategies are working and what could be improved is to ask the students who are experiencing the full breadth of the curriculum rather than depending on the snapshot view obtained when querying clinical teachers. Current literature focuses on student and faculty perspectives on specific interventions with only limited research on student experiences with and thoughts about longitudinal communication skills curriculum that spans the pre-clinical and clinical years.

The majority of U.S. medical schools teach these skills during the pre-clinical years, with little reinforcement either through formal classroom sessions or more informal clinical teaching during the clinical years [3, 5]. This disconnect between preclinical and clinical communication skills teaching has been noted as potentially resulting in the deterioration of students’ communication skills over the course of their education [3, 5–7]. Even though some schools have incorporated formal sessions on communication skills during clinical training, the lack of emphasis in informal encounters with clinical supervisors risks undermining preclinical clinical skills learning. It has been suggested that an important component of learning during clinical years is observation of learners’ interviewing skills. Incorporation of simulated patient interviews in the clinical curriculum, primarily through objective structured clinical examinations, may reflect one effort to address previous lack of observation of skills during clinical training. However, these simulated interviews have been noted as being different from interviews with real patients, both in terms of patient behavior and student approach [8]. As a result experts have postulated that an important approach to reinforcing and enhancing communication skills learned pre-clinically is through *direct* observation of medical students conducting interviews with *real* patients by supervising faculty or residents, with subsequent feedback regarding strengths and weaknesses [9–13].

A number of studies have examined the prevalence of observation in clinical learning and noted that it is typically infrequent [12, 14–17]. The value of these observation events comes from the feedback that follows them. Studies have explored feedback provided to learners during clinical rotations, noting that students appreciate it as a guide for learning but identifying that, the frequency, content, and quality of feedback can vary widely [14, 18–22]. Two things appear to be missing from the current literature: First, very few studies have looked at the quality of communication skills observation and feedback together. Second, students’ perspectives of observation and feedback regarding communication skills with real patients have not been examined. Student experiences with the teaching and evaluation of clinical communication skills has been found to greatly affect their perception of the importance of such skills in the practice of medicine [2, 23, 24]. While the idea that observation occurs infrequently and feedback provided medical learners is often inadequate has been previously noted, we were unable to find empirical research examining student perceptions of these two aspects of communication learning. The purpose of the current study is to examine student perspectives on their experiences with observation and feedback related to communication skills in order to provide a deeper understanding of their clinical learning experiences and the value they place on learning communication skills in this context.

## Methods

All 4th year students from the graduating classes of 2009/2010 and 2014 at a large Midwestern medical school were asked via email to participate in a study focusing on how they learn about communication during clinical clerkships. Students were invited to participate in one-to-one, in-depth interviews conducted by a single medical student that asked about what they found helpful in learning to interview, if and when they were observed, and what kind of feedback they had received regarding interviewing. Interview questions were piloted with five students prior to using these methods with the study cohort, and the wording was adjusted as needed to clarify questions (Additional file 1: Table S1). These questions were asked of all students interviewed with slight variations in order and additional exploration of topics as needed.

Interviews were audio recorded and then transcribed verbatim and compiled in an Nvivo8 database for thematic coding. All authors (a medical student and two non-clinicial PhDs) participated in analysis of transcripts and followed the “editing style” approach described by Miller and Crabtree [25]. All interview excerpts related to observation and feedback were read and key themes and passages were identified. After discussion, an initial list of codes was developed based on questions from the interview template and themes that commonly arose in excerpts. In the second stage, all excerpts using the initial coding listed and the coded excerpts were reviewed, and any discrepancies were discussed until consensus was achieved regarding the code list and emergent themes. The coding list was reorganized to reflect the main themes of student discussions of observation and feedback. All excerpts were then reviewed to ensure that the main themes adequately captured the range of student responses.

## Results

A total of 125 students participated in interviews that queried their experiences learning interviewing techniques and communication skills both pre-clinically and during clinical rotations. Our main focus in the current study was the subset of comments regarding direct observation by faculty/residents and feedback after these observations. The broad majority of student comments fell into one of four theme categories: 1) frequency of direct observation, 2) frequency and timing of feedback following observation, 3) content of feedback, and 4) student perceived value of the observation and feedback process. Below we explore each of these themes, providing sample comments from students that represent common perspectives discussed in the interviews.

### Frequency of observation

The majority of students reported rarely being observed. Students indicated that, in the context of the dozens of patient interviews they conducted over the course of each rotation, being observed only a few times over the course of a year felt inadequate. The majority of observation events were during specific clerkships (internal medicine, psychiatry, and obstetrics/gynecology) that required evaluation of student clinical skills with real patients as part of the rotation grade. As one student stated, “*unless it’s required, it’s not really performed often*.” Students reported being observed rarely, if ever, on other rotations. As summed up by two students:

- *[I was observed] not that often and usually it’s because you have to get something signed off on or something like that. They [faculty/residents] usually don’t [observe you] if they don’t have to.*
- *I also don’t feel like I’m watched nearly enough in my interactions with patients. You know, because otherwise I could go through a 4-week rotation and never have anybody watch me, and never know if I’m, like, asking the right questions…or if it would be better to ask them a different way.*

Students identified limited faculty/resident time as the most common obstacle to being observed:

- *Sometimes when you want to feel like a team player and you’re thinking that, of all the things that need to be done today someone watching me isn’t important, still having that requirement makes you ask.*
- *It was a very special time which the attending had to cut out of their day to watch me [interview] and you could tell and it’s not something they’re going to do every day.*

Observation of interviewing was not commonly seen as a high priority and often became a last-minute requirement that had to be completed:

- *I think my experience has been that more people have put it off towards the end of the rotation and then maybe when it does [happen] it’s done in a more hurried fashion because you’re running out of time and you have to get it done.*

Thus, while some rotations required observation of students, student perceptions were that these observations were not necessarily valued or conducted in a thoughtful manner by faculty/residents.

### Frequency and timing of feedback

Opportunities to receive feedback on communication skills were severely limited by the infrequent nature of observation, and even when students were observed, they did not always receive feedback.

- *If you’re doing something blatantly wrong the residents will usually tell you, but other than that they don’t really say much.*
- *Usually they said “good job” or something like that. I don’t think I was ever given something to work on.*

Because most observation events were formal observations required to complete the rotation, many students did not receive associated feedback until their grade was determined at the end of the rotation, often days to weeks after the observed interview. This limited the value of the feedback because the student often couldn’t remember the exact interview that was observed.

- *I think it’s helpful if…they can give you immediate feedback. It’s really hard, again it’s the time crunch thing, but if possible to do that because sometimes neither one of you remembers that interview very well. So if it can happen sooner, the better, even if it’s later that day or at the end of the week, instead of 3 weeks or 6 weeks [later].*

Some students reported having to explicitly ask for feedback. They often got useful information after asking but stated they often felt uncomfortable having to request feedback.

- *[Sometimes you have to] just ask the resident or the attending, and they’ll tell you, so sometimes it’s just a matter of the student being more proactive in their education and being like, ok, ‘What was good about it?’ And that can be scary, but when you ask, you get good constructive feedback.*

### Content of feedback

The content of feedback students reported receiving also varied widely. Many students stated the feedback they received was generally vague and unhelpful:

- *It’s always more helpful when the person who’s observing gives constructive feedback rather than just, ‘That was good,’ and that’s it.*

When students were given specific feedback, it often focused on information-gathering rather than interpersonal/communication skills:

- *I think there was one that maybe said something about the way that I interacted but they usually don’t give that much feedback…The feedback hasn’t been that great, it’s usually covering “I would have asked this” but not the interaction itself.*

Feedback about communication skills ranged from vague or overly specific:

- *When they do talk about it, it’s usually a quick one line of saying, ‘Oh, you did a good job establishing rapport,’ and then they move on and ask about the information that I actually gathered.*
- *…sometimes [observers would give] off the wall critiques of small things that would then become the focus of the feedback. And so, instead of the emphasis on, ‘you did these things very well, build on those and work on this,’ it was ‘you said Mrs. Jones instead of Barbara when you came into the room.*

### Value of observation and feedback

A majority of students felt that being observed interviewing and receiving feedback was helpful in guiding them to becoming efficient and effective interviewers and communicators. As discussed above, the number of observation events was often very low and students consistently expressed a desire for more opportunities:

- *I wish people watched me [interview]. Because I think when I do have a professor that actually does take the time to give me feedback, it’s amazing how much I can learn from that person. And I don’t think we get enough feedback.*
- *It’s very frustrating because otherwise you are just doing what you think you need to do but you never get any feedback, and any feedback that you get is going to be conjecture. I really feel like having more direct observation and feedback by faculty would be immensely helpful.*

While students did report getting feedback on their presentations, they felt this was not an accurate way to assess their communication skills and interviewing technique.

- *How you’re wording things, maybe the order that you’re doing things in and subtleties like that don’t come across when you present the patient. Because I usually get pretty good remarks in presenting my patients because I’ve had time to sit and re-create the story in a logical way, but I think if some of those staff that gave me good feedback on my presentations saw me obtaining that information, I think they might have something else to say. Just because in my head it’s still…very random and messy and jumping around a lot. So in that way I think that presenting, while it does give me good feedback, isn’t really an accurate reflection of the actual interview itself.*

Some students, however, did not see a benefit in being observed. They felt they learned more from trial and error and sometimes saw being observed as an indication of incompetence:

- *Some students might need a resident with them all the time to watch them because they struggle in that area but other students don’t really need a resident to watch them just because you know what you’re doing. I think the resident gets a sense of that pretty early, because if they don’t have to sit there and watch you they’re stoked about that because then they can do their work and they can trust you to do a good job. I think if residents don’t have to watch you, they don’t want to watch you.*

A common theme among students was that being observed, while ultimately helpful, was uncomfortable and even potentially changed the way they interviewed patients:

- *…as much as it helped I feel like it was unnatural and a little bit forced.*
- *I’d just get nervous and wouldn’t do as well as I thought I would have done if somebody hadn’t been watching over my shoulder.*
- *You’re focusing on the patient but out of the corner of your eye you’re always seeing the resident, how they’re reacting to your questions, if they’re getting agitated because you’re talking too long.*

Students were often worried about conducting the interview in the “right” way because most observed patient encounters under the current curriculum are required, formal evaluations using formal assessment instruments such as the Mini-CEX, a standardized form which primarily utilizes a numbered scale to assess student clinical skills [26]. Learners perceived this as shifting the focus of the interview from the patient to the grade. Even in situations where the Mini-CEX was not used to calculate a final rotation grade, the mere act of being scored on a numerical scale changed students’ approach to the interview. Students suggested uncoupling observation from grading and increasing the number of observations to make students more comfortable:

- *I think it could be done…under more informal situations…like having [faculty/residents] go in with you like once a week or something…and have them critique you. Instead of having them hold a paper and, like, check off things as you’re asking the questions.*
- *I think that for lots of students just getting comfortable … is really hard. And so I really think…the more low stress ways that we can practice but still get feedback, the better.*

While students brought up several concerns regarding the observation process and the feedback they received, they did point to examples where observation and feedback were helpful in their learning.

- *When someone watches you who does it all the time, and has been doing it for a long time, they pick up on stuff that you wouldn’t necessarily think of.*
- *I’ve learned a lot about myself, the way I interview or ask questions from those feedback sessions, potentially things that I didn’t realize before.*
- *They talked about how I responded to the patient’s concerns, how I created rapport with the patient, asking open-ended questions, summarizing.*

Some supervising physicians were identified as being very effective in giving communication skills–focused feedback, using terms students had learned in the first 2 years of medical school to discuss their clinical interviewing. Students valued the experience residents and attending physicians brought to the observation and feedback process and saw the benefit that third-party feedback can add to learning compared to self-reflection alone.

## Discussion

The purpose of this study was to explore student experiences with and perspectives on observation and feedback on their communication skills with patients during clinical training. As noted, previous studies have explored student perspectives on either observation or feedback related to communication skills during clinical training and have not addressed them together. The current study explored both observation and feedback related to communication skills, therefore providing valuable information on student perspectives regarding how to improve their own communication education.

The main findings of this study are that students felt they were directly observed by faculty and/or residents infrequently and reported receiving feedback regarding their communication skills even less frequently. The majority of students perceived this method of instruction helpful in developing their interviewing skills and expressed a desire for more opportunities for observation and feedback. While observation and feedback has the potential to reinforce and refine students’ communication skills, the significance of this study is in its identification of a number of issues that could be addressed in order to increase the effectiveness of the observation and feedback process as a tool for student communication learning.

One of the most obvious issues is simply the lack of observation events and therefore opportunities for feedback. Only a small percentage of patient interviews conducted by students were observed, and not all of those observation events resulted in feedback. This finding is similar to other studies that have explored observation of students during clinical clerkships [16, 22]. For example, in a survey of 3rd year medical students, the majority reported never being observed by faculty in patient interviews [17]. The current study found that when feedback was received, rather than centering on specific communication skills it instead was more often focused on the content of questions asked and information gathered. Thus, a model of this phenomena can be visualized as an upside-down pyramid in which students conduct many interviews with real patients, few of which are observed, even fewer in which feedback is provided, and even fewer with feedback specifically directed at communication skills.

A large obstacle that prevents students from getting the maximum benefit from post-observation feedback is the discomfort students feel when being observed. This phenomenon was commonly linked with both the infrequency of observation and the graded nature of most observation events. It should be noted that the suggestion that formal evaluations are an obstacle to meaningful feedback stands in direct opposition to much published literature that emphasizes the utility of various workplace-based assessments (WBAs), for example, the mini-CEX, as tools for student education [2, 9, 10, 19, 27]. The current study identifies student perceptions of what is at stake; while requiring observation undoubtedly increases its frequency, the pressurized environment created by correlating it with a student’s final grade changes the focus from conducting quality interviews in the present to academic standing and career aspirations in the future. The tenuous balance between grading and education has been noted in the context of WBAs but never with a specific focus on communication skills, which must be observed directly in order to generate any meaningful feedback [28–32]. Even when the individual-scored observation event was not used to calculate the student’s final grade, the use of a numerical rating led students to focus on how that rating would be reflected in their final evaluations. Importantly, students in this study stated that they not only changed what content they elicited during an observed interview, but that they changed the process by which they did it. It should also be noted that the current Mini-CEX tool used by the majority of rotations at this institution has only one item out of ten that pertains to communication skills and therefore does not offer much of an impetus to give meaningful feedback on communication skills in particular.

One potential strategy to address student discomfort suggested by some of the students in our study would be to increase the number of informal, ungraded observations to both increase student comfort and lower the stakes associated with observation with a focus on rotations that currently observe less frequently than other rotations. This would be in keeping with more general recommendations in the literature that to be an effective learning tool focused on learner improvement, feedback (formative evaluation) should be distinct rather than equivalent to summative evaluation [14, 18, 22]. Implementing more required observations is easier said than done, however, due to the limited time (real or perceived) faculty and/or residents have available to observe students interviewing. It is commonly acknowledged that most physicians are spending less and less time with each patient, and students are acutely aware of the many demands on their supervisors’ time. Students often see their own training as low on the list of priorities, but it is unclear whether this is explicitly expressed by their supervisors or an idea perpetuated by students [2]. Faculty development to help supervisors to develop time-efficient ways to incorporate observation into their daily clinical duties would help address this issue. As an example, Lane and Gottlieb successfully trained faculty to conduct brief observations (3–4 min) and provide focused feedback to students on a pediatrics rotation, resulting in a significant increase in the number of times students were observed during the rotation [33].

The major issue associated with the timing of feedback post-observation was the delay between the observed interview and the receipt of feedback. Students often had to wait weeks to receive feedback, and this severely limited the benefit they reported getting. Again in keeping with the general literature on feedback, a strong push should be made for observers to give their feedback in a timely manner, preferably as soon as possible [15, 19, 34–36]. A verbal debriefing after the observation could greatly help guide students to make immediate and beneficial changes in their interviewing technique and communication skills. Students should also be encouraged to ask their observers for feedback whenever they think it would be helpful. Students often feel that such a request may be seen as imposing or annoying, but normalizing the practice could greatly empower students to be more proactive in their own education.

The content of feedback, when students received it, was often vague and largely centered around gathering medical information and forming diagnostic questions (the content) rather than communication skills (the process). Both faculty and residents could benefit from faculty development focused on helping observers with both their observation skills as well as how to give behaviorally specific, focused feedback and use communication terms that students had previously encountered in their curriculum. Having a working vocabulary to describe interpersonal skills would help faculty to give focused and meaningful feedback to students.

During clinical training, observing students interviewing patients is potentially one of the most effective ways to gain a clear picture of their communication strengths, as well as areas for improvement, and it provides the opportunity to make students aware of these issues in a timely manner through feedback. These communication skills need to be emphasized early because they form the basis of the doctor-patient relationship and have significant impact on patient care.

We identified several limitations in this study. The study was conducted at a single institution which tempers the generalizability of the findings. We sent invitations to participate in the study to all medical students in the classes of 2009, 2010, and 2014 who had completed their first year of clinical clerkships, but only a percentage (about 30 %) of those invited volunteered. It is likely that self-selected participation introduced some bias into the data collected and that these students were not representative of the student body as a whole. We also asked students to comment on events from up to 1 year prior, which likely introduced recall bias.

Further study could focus on real-time (in clerkship) data collection involving all medical students, limiting the sources of bias introduced by volunteer participation and recall. This approach would also offer an opportunity to collect precise quantitative data on the number of observation events as well as the frequency and quality of post-observation feedback. Follow-up studies could also be done after instituting any intervention, such as faculty development focusing on communication skills feedback or uncoupling observation events from formal evaluations, as suggested above.

## Conclusions

While the idea that learners are infrequently observed during interviews with real patients is not novel, the current study adds to our understanding of the role that observation and feedback play in the development of students’ communication skills. By focusing on the student perspective, valuable insight can be gained into what obstacles prevent students from receiving maximal educational benefit from observation and feedback, and this article suggests several means of improving this process including increasing the number of observations, disassociating observation from numerically scored evaluation, training faculty to give meaningful feedback, and timing the observation/feedback earlier in clerkships.

## Additional file

Additional file 1: Table S1. Interview Questions. List of questions used in interviews with study participants. (DOCX 86 kb)
