# Supplementary material for: ‘I wish someone watched me interview:’ medical student insight into observation and feedback as a method for teaching communication skills during the clinical years
Source: BMC Med Educ. 2016 Nov 9;16:286. doi: 10.1186/s12909-016-0813-z (PMC5103441; doi:10.1186/s12909-016-0813-z)
Supplement: Additional file 1: Table S1. — Interview Questions. List of questions used in interviews with study participants. (DOCX 86 kb) [file 12909_2016_813_MOESM1_ESM.docx]

Table 1. Interview questions.

| Please describe in detail medical interviews, both effective and ineffective, that you have observed during your clinical rotations. Include description of what about these encounters you perceived as effective and ineffective? |
| --- |
| What stands out to you about your training in medical interviewing? |
| To what extent does what you have learned and observed on clinical rotations match what you learned about medical interviewing in your Foundation of Clinical Practice courses? |
| What was effective about the way you learned medical interviewing during FCP courses? |
| How has your approach to medical interviewing changed during your clinical rotations? |
| How could the way we teach medical interviewing be improved? |
| What else do you wish you would have learned about medical interviewing before starting clinical rotations? Since starting clinical rotations? |
| Do you have any other comments about your training and experience related to medical interviewing? |
| Does the term “medical interviewing” mean the same to you as doctor-patient communication or is it something different? |
| What are the main ways you have learned about communication during your clinical clerkships? |
| If you had to teach someone how to effectively conduct a medical interview what would you teach them? How would you teach them? |
